# Customised virus‐like particles: Opening a new chapter for clinical precision gene therapy

**DOI:** 10.1002/ctm2.70395

**Published:** 2025-07-24

**Authors:** Xingyu Zhu, Qingye Zhang, Xujiao Zhou, Yujia Cai, Jiaxu Hong

**Affiliations:** ^1^ Department of Ophthalmology, Eye & ENT Hospital, State Key Laboratory of Medical Neurobiology and MOE Frontiers Center for Brain Science Fudan University Shanghai China; ^2^ Key Laboratory of Systems Biomedicine (Ministry of Education) Shanghai Center for Systems Biomedicine Shanghai Jiao Tong University Shanghai China; ^3^ Department of Ophthalmology Children's Hospital of Fudan University National Pediatric Medical Center of China Shanghai China

1

The CRISPR/Cas system, first discovered as an adaptive immune mechanism in bacteria and archaea, has evolved into a revolutionary technology capable of editing DNA loci and correcting genetic errors, offering new hope for the treatment of numerous hereditary and refractory diseases.^1^, ^2^, ^3^ This system employs a single‐guide RNA (sgRNA) to direct the endonuclease Cas9 to a specific DNA target, where it generates double‐strand breaks, thereby enabling site‐specific modification.^1^ In 2023, the US FDA approved the first gene therapy utilising CRISPR/Cas9 (Casgevy) for the treatment of sickle cell disease. This remarkable milestone has inspired researchers to continuously explore and push forward the clinical application of CRISPR‐based therapies.

The safety and efficacy of gene therapy are two critical factors that must be prioritised when applying CRISPR in vivo.^4^ Although several clinical trials – including those involving Casgevy – have demonstrated promising results, the long‐term safety and efficacy remain to be fully observed. To achieve targeted and precise delivery of CRISPR components, various engineered carrier platforms have been developed, including nanomaterials, viral particles, and exosomes.^5^, ^6^ Among these, virus‐like particles (VLPs), owing to their transient expression, efficient delivery, and low immunogenicity, have emerged as a highly promising delivery modality.^7^ However, existing editing strategies based on VLPs have thus far failed to achieve cell‐targeted gene editing in vivo.

To address this unmet need, a modular, targeted VLP platform named RIDE (RNP Integrating with Designer Envelope) was recently developed in our lab.^8^ RIDE enables cell‐selective genome editing while preserving safety and transience.

A key strength of the RIDE platform lies in its dual modularity: the use of engineered VLPs to deliver Cas9 ribonucleoproteins (RNPs) and the incorporation of customisable envelope proteins for cell‐specific targeting. Unlike conventional delivery systems that rely on passive diffusion or broad viral tropism, RIDE has been shown to be rationally redirected to virtually any cell type, either by adapting to the local tissue environment or by employing engineered ligands such as single‐chain antibodies or DARPins. This flexibility represents a conceptual shift from broadly systemic, persistent delivery toward transient, tunable, and cell‐selective genome editing – an essential step toward realising personalised and tissue‐specific gene therapies.

Complementing its targeting versatility, RIDE also features a transient mode of action. Traditional vectors, such as adeno‐associated viruses (AAVs) or lentiviruses (LVs), rely on DNA‐based delivery, which can lead to prolonged Cas9 expression and increase the risk of off‐target effects and genomic instability. In contrast, RIDE delivers Cas9‐sgRNA RNPs directly into target cells, ensuring a short intracellular half‐life and transient editing activity. This approach significantly reduces the risk of insertional mutagenesis and cytotoxicity while maintaining robust in vivo editing efficiency.

Importantly, by avoiding the introduction of foreign DNA, RIDE enhances biosafety and meets critical regulatory standards, thus positioning it as a promising delivery platform for clinical genome editing. As genome editing approaches transition toward therapeutic applications, RIDE offers a safer and more precise strategy that may reshape the future of gene therapy.

In addition to its safety and efficacy, RIDE offers further advantages with its immunological profile. One major limitation of current CRISPR delivery platforms – particularly those based on AAVs or LVs – is their immunogenicity, which restricts repeat dosing due to humoral responses against Cas9 or vector components. RIDE addresses this issue by delivering Cas9 as a transient RNP complex, thereby minimising antigen persistence. In both murine and non‐human primate models, RIDE elicited minimal innate immune activation and, notably, did not induce detectable anti‐Cas9 IgG antibodies. While low‐level anti‐p24 responses were observed – consistent with the VLP origin – immune cell infiltration was markedly reduced, particularly in immune‐privileged tissues such as the eye. Collectively, these findings underscore RIDE's favourable immunological profile and its suitability for repeated administration – an essential requirement for certain conditions due to the need for widespread systemic delivery or the short lifespan of target cells (Figure 1).

**FIGURE 1:**
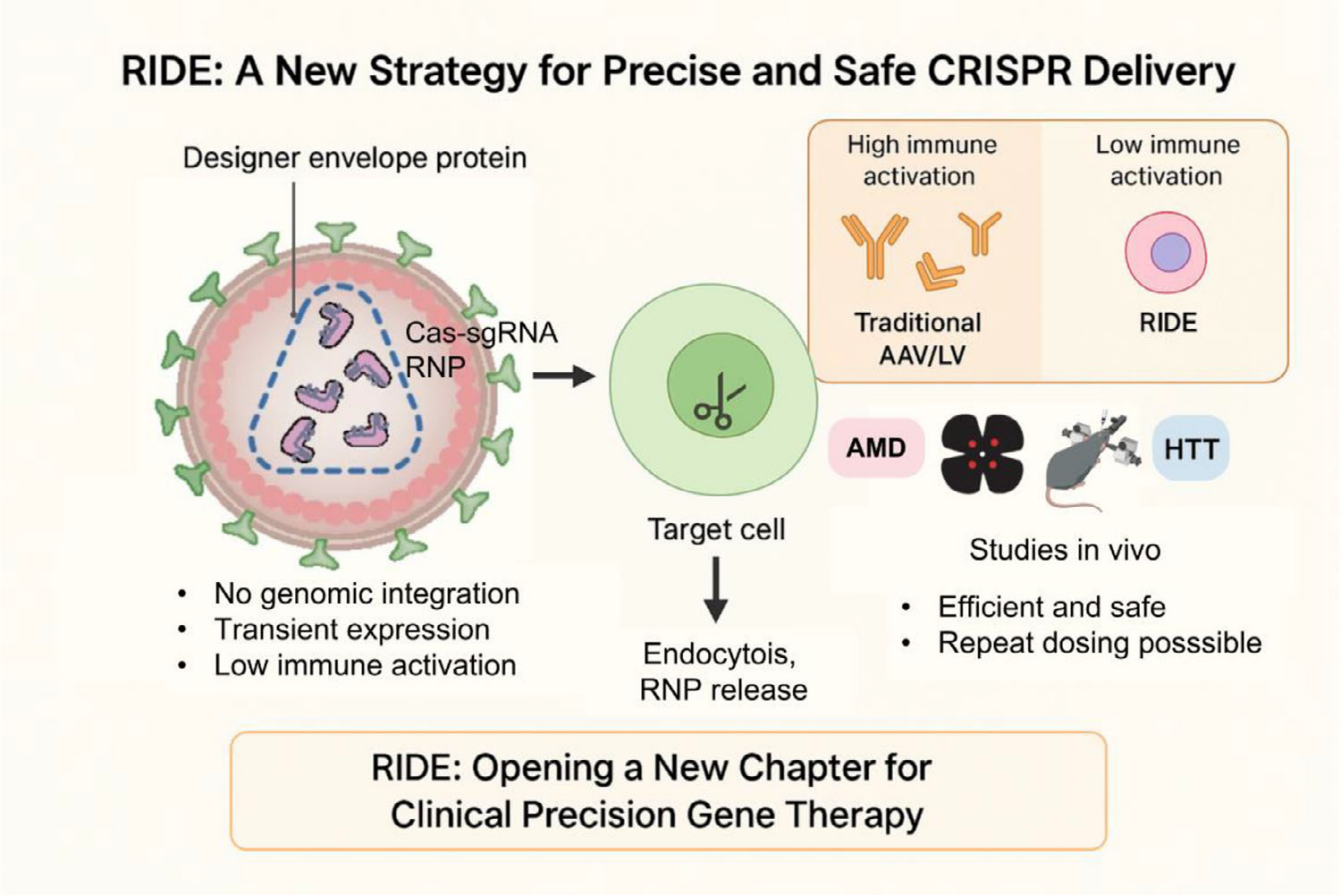
Schematic overview of the RIDE (RNP Integrating with Designer Envelope) system for CRISPR‐Cas9 delivery.

To further assess RIDE's translational potential, in vivo studies were conducted targeting the eye and central nervous system – two representative immune‐privileged sites. These experiments demonstrated robust and localised gene editing in preclinical models of retinal vascular disease and Huntington's disease, highlighting RIDE's capacity for therapeutic precision without prolonged transgene expression.

Surprisingly, however, even within the relative immune sanctuary of the eye, single‐cell transcriptomic analyses revealed that RIDE‐mediated editing triggered infiltration of both innate and adaptive immune cells, along with epithelial‐to‐mesenchymal–like transitions in retinal pigment epithelial (RPE) cells. These responses, though mild, suggest that even transient editing events may not be entirely immunologically silent.

These findings challenge the prevailing assumption that non‐integrating delivery platforms are immunologically inert and instead reveal the complex immunobiology surrounding genome editing – including host responses not only to the delivery vector but also to the editing event itself. As genome editing expands into developmentally sensitive tissues and stem cell–derived organoids, these observations underscore the importance of high‐resolution immune and cellular monitoring to ensure long‐term safety. While RIDE's modularity and targeting precision offer a compelling platform, its clinical application must be matched by equally rigorous tools to map host responses in fragile physiological contexts.

Although studies to date have focused on localised delivery to immune‐privileged sites such as the eye and brain, the design of RIDE also supports broader systemic applications. Its transient, non‐integrative nature and modular assembly render it particularly well‐suited for targeting widely distributed cell populations, such as hematopoietic or immune cells, where durable expression is neither required nor desirable.

This potential is already beginning to be realised. During the preparation of this manuscript, Hamilton et al.^9^, ^10^ independently reported in vivo gene editing of T cells using VLP‐based delivery, reinforcing the feasibility of RNP‐mediated systemic editing. As genome editing advances from proof‐of‐concept to clinical practice, platforms like RIDE – capable of balancing precision, safety, and flexibility – may prove instrumental in extending therapeutic applications beyond localised diseases to complex, multi‐organ disorders.

RIDE represents more than a technical advancement – it signifies a conceptual leap in genome editing delivery. By decoupling CRISPR activity from persistent expression and enabling modular, cell‐specific delivery with minimal immunogenicity, RIDE sets a new standard for clinical‐grade genome editing. Its transient and programmable nature not only expands the therapeutic landscape to include immune‐privileged or inflamed tissues but also reinvigorates essential discussions on precision, redosability, and safety. As the field moves toward clinical implementation, platforms like RIDE will be pivotal in translating molecular innovations into real‐world therapeutic solutions.

## AUTHOR CONTRIBUTIONS

X. Zhu and Q. Zhang conceived and wrote the article. All authors contributed to the additional writing and editing of the article.

## CONFLICT OF INTEREST STATEMENT

The authors declare no conflict of interest.

## ETHICS STATEMENT

Our study did not involve animal and human clinical trails and was not unethical.
